# Integrated water quality assessment and health risk analysis of heavy metal and microbial contamination in the Ichu River, Peru

**DOI:** 10.12688/f1000research.162022.2

**Published:** 2025-10-06

**Authors:** Cesia Rebeca Zarate-Cáceres, Melva Iparraguirre-Meza, Claris Jhovana Pérez-Venegas, Juan Antonio Picoy-Gonzales, Gabriela Ordoñez-Ccora, Pavel Lacho-Gutiérrez, Kelly Yadira Riveros-Laurente, Diana Lizeth Diaz-Aranda, Geovanna Geraldine Gutiérrez-Iparraguirre, Rosaura Huarcaya-Taype, Rocío Paula Arias-Rico, Yda Flor Camposano-Cordova, Lida Ines Carhuas-Peña, Arnaldo Virgilio Capcha-Huamani, RUSSBELT YAULILAHUA HUACHO

**Affiliations:** 1 Facultad de Ciencias de la Salud, National University of Huancavelica, Huancavelica, 09001, Peru; 2 Facultad de Ciencias de la Salud, Universidad Peruana Los Andes, Huancayo, Junin, 12002, Peru; 3 Facultad de Ciencias de Medicina, Universidad Nacional Mayor de San Marcos, Lima District, Lima Region, 15081, Peru; 4 Facultad de Ciencias de Ingeniería, National University of Huancavelica, Huancavelica, Huancavelica, 09001, Peru

**Keywords:** Water pollution, heavy metals, microbial contamination, risk assessment, Ichu River

## Abstract

**Background:**

The Ichu River serves as the primary water source for urban and agricultural use and industrial operations, but anthropogenic pollution has a serious negative impact on its water quality.

**Methods:**

The investigation measured water quality and health-related risks by analyzing physicochemical parameters, heavy metals, and microbial pollutants at eight sampling points, site 1 (S1) through (S8).

**Results:**

The research data showed that water quality worsened progressively from upstream to downstream locations such as turbidity, TDS, conductivity, and BOD levels increased. Oil pollution and oxygen depletion arose from a reduction in dissolved oxygen from 6.3 to 4.5 mg/L at the different sampling sites (S1 to S8). Heavy metals (As, Pb, Cd, and Cr) in the samples exceeded the standards established by the World Health Organization (WHO) established standards because of mining and industrial wastewater and local wastewater discharge. The presence of excessive *Escherichia coli* (E. coli) and total coliforms in microbial tests proved that the water was severely contaminated by fecal matter. Principal Component Analysis showed that heavy metals exist with microbial pollution and organic load as the main sources of water quality decline, and pollution indicators were found to establish powerful relationships with depleted oxygen levels.

**Conclusion:**

The severe contamination risks found in this study justify immediate pollution control measures, wastewater treatment enforcement, and sustainable watershed management practices. Urgent action is necessary because vital parameters surpass the standards set by the WHO and (United States Environmental Protection Agency (USEPA) to avoid enduring environmental damage and health problems. This research demonstrates the value of continuing water quality assessments while enforcing policies and raising public awareness to improve the water quality of the Ichu River.

## Introduction

The existence of water as a resource plays a crucial role in sustaining human life, together with economic activities, and maintaining ecological balance. Water quality has suffered a severe decline because of escalating industrial expansion combined with agricultural runoff and growing domestic waste deposition ( Saxena, 2025; Edo et al., 2024). The contamination of rivers presents a fundamental issue because rivers act as the main waterfront supplies that people use for drinking water, irrigation, and domestic purposes. The Ichu River in Peru faces two serious threats to its ecosystem and human health: heavy metal pollution and microbial contamination ( Sánchez-Araujo et al., 2024). Water quality assessment, combined with health risk evaluation for river contaminants, must be performed because of the region's economic reliance on this water source.

Heavy metals, including arsenic (As), lead (Pb), cadmium (Cd), mercury (Hg), and chromium (Cr), found in river water are of major concern because they are non-degradable in the environment and tend to accumulate in living organisms ( Rahman and Singh, 2019; Khalef et al., 2022). Toxic elements cause neurological disorders, kidney damage, and carcinogenic effects in humans, despite minimal exposure levels. Contamination of the Ichu River arises from mining practices, along with industrial waste discharge and natural geological factors that harm water quality conditions while posing threats to human health. Such vital assessments of chronic daily intake (CDI), hazard quotient (HQ), and carcinogenic risk (CR) allow scientists to determine exposure levels and potential health disorders. Studies have shown that rivers within mining-affected zones exceed established heavy metal safety limits for drinking water set by the World Health Organization (WHO) and the United States Environmental Protection Agency (USEPA) for drinking water ( Custodio et al., 2020; Kumi et al., 2023: Yin et al., 2024).

Recent research has demonstrated that riverine ecosystems worldwide are increasingly threatened by nutrient enrichment, heavy metal accumulation, and microbial contamination, largely linked to rapid urbanization and industrial discharges ( Singh et al., 2021; Choudhury et al., 2022). Studies from the Hindon River in India ( Singh et al., 2024a), the Ganges basin ( Li et al., 2022), and Latin American river systems ( TQEM, 2023) show that water quality decline is a shared challenge across regions. These references reinforce the scientific justification of the present study.

Globally, poor water quality has been linked to biodiversity decline, increased ecological risk, and heightened public health concerns ( Gaur et al., 2022; Singh et al., 2024b). Regionally, rivers in South Asia such as the Yamuna and Hindon, and in Latin America such as the Mantaro, demonstrate similar patterns of degradation, often leading to unsafe irrigation water, reduced fisheries, and waterborne diseases ( Choudhury et al., 2022; Singh et al., 2024a). These findings underscore the urgent need to monitor and manage water resources systematically.

Heavy metals are not the only severe pollution problem that burdens the Ichu River because microbial contamination poses additional threats ( Muze et al., 2020). The occurrence of Escherichia coli (E. coli) along with total coliform bacteria indicates fecal pollution, which generally stems from untreated sewage outlets and agricultural pollution together with livestock waste. High microbial pollution levels in water create substantial disease risks that primarily endanger children and elderly individuals through conditions including diarrhea, dysentery, typhoid, and cholera. Quantitative bacterial evaluations performed using the Most Probable Number (MPN) method enable measurements that help identify whether river water qualifies as drinking water and domestic water sources ( Chigbu and Sobolev, 2007; Brand, 2012). For example, the Yamuna River in India exhibits persistent organic and metal contamination due to untreated sewage ( Singh et al., 2021), while China’s Yellow River suffers from severe industrial effluent discharge ( Su et al., 2021). In South America, Peru’s Mantaro River faces similar pressures from mining and agriculture ( Jyethi et al., 2022). Such case studies highlight the global relevance of local river quality assessments. The research initiative integrates the assessment of water quality through combined physicochemical evaluation, heavy metals, and microbial contamination analysis of the Ichu River system. This research assesses potential health risks for local people using health risk assessment models to determine both non-carcinogenic and carcinogenic effects. The study measures water quality levels against WHO and USEPA standards to identify major pollution sources and present viable control measures. This research provides essential references to local authorities, environmental agencies, and policymakers for creating sustainable water management approaches with public health protocols.

The principal significance of this study lies in providing the first integrated assessment of the river’s physicochemical and biological quality. Beyond generating baseline data, the findings contribute to ecological risk assessments and inform sustainable water management strategies in the region.

## Methods

### Study area and sampling sites

Community members in Peru depend on the Ichu River for their freshwater needs because they supply drinking water, as well as for both farming and industrial applications. Little human activity occurred at Sampling Site 1 (S1), which serves as the baseline reference site situated within the remote upper region of the river. Semi-urbanized locations that contained industrial and agricultural activities were selected as Sampling Sites 2 to 4 (S2–S4), thus contributing to possible water pollution. The district receives effluents from mining activities and untreated sewage and industrial wastewater discharges between Sampling Sites 5 and 8 (S5–S8) located in this area. All sampling site locations have been provided below as S1, S2, S3, … , corresponding to the different river stretches covered in this study. Photographic illustrations of these sites highlight the surrounding land use and visible pollution sources, offering clearer context for the environmental conditions observed.

• **S1 (Upper Ichu River)**: Remote headwater region with minimal human activity (baseline reference site).
• **S2 (San Jerónimo)**: A semi-urbanized area with agricultural and small-scale industrial activities.
• **S3 (Yauli)** – Agricultural zone with increasing human settlements and irrigation use.
• **S4 (Ascensión)** – Transitional area with mixed urban, agricultural, and industrial influence.
• **S5 (Huancavelica)**: A major urban center receiving wastewater from residential and commercial activities.
• **S6 (Santa Ana)** – Industrialized district with significant mining and manufacturing effluents.
• **S7 (Acobambilla)**: Downstream rural area affected by wastewater and surface runoff contamination.
• **S8 (Vilca)**: Lower basin region experiencing cumulative pollution impacts from upstream sources.

### Sample collection and preservation

The polyethylene bottles were cleaned prior to use as containers for physicochemical tests and heavy metal evaluations, whereas glass bottles served as sterile containers for microbial analyses. The procedure for avoiding contamination included rinsing all bottles with river water immediately at the sampling site before specimen collection. A depth of 30 cm beneath the water surface served as the sampling point because scientists wanted to bypass atmospheric influences. Physicochemical and heavy metal samples were stored in ice boxes set to 4°C before laboratory analysis was performed. The laboratory examined microbiological samples no later than six hours after their collection time to stop bacterial breakdown. To stop heavy metal adsorption or precipitation during the analysis, the samples were treated with nitric acid (HNO _3_, pH < 2) (Sigma-Aldrich, 21641-1L). The laboratory followed the American Public Health Association standard methods for all sampling and preservation stages to establish consistent and reliable testing procedures.

### Physicochemical analysis

Water samples underwent physicochemical testing through field instruments and laboratory methods to measure pH with a Hanna Instruments digital pH meter (USA) and turbidity with a Hach 2100P turbidimeter alongside TDS and EC analysis using a YSI 556 MPS multi-parameter probe (USA). The multi-parameter probe YSI 556 MPS (USA) measured the TDS and EC to evaluate the dissolved ion concentrations within the water samples. The Extech DO600 oxygen meter enabled DO measurements in the water column, and the 5-day incubation method at 20°C allowed the determination of BOD _5_ according to APHA Standard Method 5210 B. Researchers used UV-Vis spectrophotometry to measure nitrate (NO _3_^-^) and phosphate (PO _4_^3-^) concentrations because these chemicals indicate agricultural runoff pollution. All measuring tools underwent pre-use calibration to generate reliable and accurate data outputs. The National Sanitation Foundation Water Quality Index (NSF-WQI) and Canadian Council of Ministers of the Environment Water Quality Index (CCME-WQI) were calculated to standardize the interpretation of physicochemical parameters. These indices are widely used for comparing water quality across regions ( Su et al., 2021; Singh et al., 2021).

### Heavy metal analysis

The analysis focused on measuring arsenic (As), lead (Pb), cadmium (Cd), mercury (Hg), and chromium (Cr) in water samples using Inductively Coupled Plasma Mass Spectrometry (ICP-MS, Agilent 7500 Series, USA). The acid digestion required treating 50 mL of water sample solution with HNO _3_ under 95°C heat for two hours before organic matter breakdown. ICP-MS analysis of filtered and diluted digestions took place through 0.45 μm membrane filtration. The analysis procedure adopted Certified Reference Materials (CRMs, NIST 1643f ) for instrument calibration and required multiple runs for each sample to verify the consistency of the results. The interim procedure included the execution of blanks and standard solutions to prevent sample contamination between tests. Heavy metal concentrations in tap water samples were measured to evaluate their compliance with the WHO drinking water quality standards as well as the USEPA limits.

### Microbial contamination analysis

The Most Probable Number (MPN) method (APHA Standard Method 9221 B) allowed for the assessment of microbial contamination through E. coli and total coliform enumeration. Each water sample received 10 mL, 1 mL and 0.1 mL aliquots which were inserted into Lauryl Tryptose Broth (LTB) tubes before being placed at 35°C for 24–48 hours of incubation. Tubes producing gas signified a presumptive positive result, and post-confirmation transfers occurred in Brilliant Green Lactose Bile (BGLB) broth. The researchers counted positive tubes to determine microbial concentrations within the water samples using the Most Probable Number tables. Sterile glassware and autoclaved media maintained the reliability of microbial results throughout the entire process. Regular testing was performed twice for every sample, and control bacterial cultures of E. coli ATCC 25922 were included as part of the testing procedure. The data from the tests were cross-referenced with the WHO drinking water standards, where E. coli and total coliforms must not appear in drinking water (0 MPN/100mL).

### Health risk assessment

To evaluate the potential health risks associated with long-term exposure to heavy metals, three key risk assessment models were applied: Chronic Daily Intake (CDI), Hazard Quotient (HQ), and Carcinogenic Risk (CR).

The CDI (mg/kg/day) was calculated using the following equation:

CDI=C×IR×EF×ED\BW×AT

where C represents the metal concentration (mg/L), IR is the ingestion rate (2 L/day for adults and 1 L/day for children), EF is the exposure frequency (365 days/year), ED is the exposure duration (70 years for adults and 6 years for children), BW is the body weight (70 kg for adults and 15 kg for children), and AT is the averaging time (ED × 365 days).

Non-carcinogenic risk was assessed using the Hazard Quotient (HQ) formula:

HQ=CDI\RfD

where RfD (mg/kg/day) is the reference dose per the USEPA guidelines. An HQ value greater than one indicates a potential health risk. The Carcinogenic Risk (CR) was estimated using:

CR=CDI×SF

where SF is the cancer slope factor (mg/(kg · d)) provided by the USEPA. A CR value exceeding 1E-04 is considered a significant risk factor for cancer.

### Statistical analysis and data Interpretation

The research data were statistically analyzed using IBM SPSS v26 and Microsoft Excel for processing. All parameters received descriptive statistical treatment using the mean and standard deviation assessment. A Pearson correlation test was used to examine the relationship between water quality and heavy metal content, as well as microbial contamination levels. Principal Component Analysis (PCA) used to detect the main pollution sources affecting the Ichu River.

## Results

### Physiochemical properties of Ichu River across various sites

A range of physicochemical parameters from the Ichu River showed considerable variations among sampling stations S1 through S8, as human activities such as industrial waste release, agricultural water flows, and uncontrolled domestic waste contributed to these changes. The standard deviation (±σ) represents the mean values used to measure both the natural variability and measurement uncertainty in the study results. The pH measurements at the upstream site (S1) amounted to 7.9 ± 0.41 while the downstream site (S8) recorded a value of 6.7 ± 0.41 ( Table 1). This downward trend in pH likely results from industrial and domestic wastewater emissions. Observational data show that the most basic condition exists at S1, where pH indicates neutral conditions, whereas S8 exhibits the lowest reading, indicating that the pollution effect leads to acidic conditions. The downstream water bodies showed higher levels of sediment and organic matter as turbidity values increased from 2.8 ± 1.95 NTU (S1) to 7.9 ± 1.95 NTU (S8). The possible contributors include erosion, agricultural runoff, and sewage discharge. The examination of the total dissolved solids showed rising numbers from 41.2 ± 5.24 mg/L (S1) to 54.8 ± 5.24 mg/L (S8) because of increased dissolved pollutants and minerals during the journey towards the river's lower section. The pollution from organic substances resulted in a reduced DO concentration ranging from 6.3 ± 0.62 mg/L (S1) to 4.5 ± 0.62 mg/L (S8) ( Table 1). The organic matter decomposition increased in the lower reaches as shown by the BOD levels which rose from 14.8 ± 4.61 mg/L (S1) to 28.5 ± 4.61 mg/L (S8). The water temperature decreased steadily from 22.5 ± 0.58°C (S1) to 20.8 ± 0.58°C (S8) when considering seasonal changes alongside shading effects. The water samples collected at S1 had 3.2 ± 0.84 mg/L NO _3_^-^ while S8 samples showed 5.5 ± 0.84 mg/L NO _3_^-^ and 0.21 ± 0.10 mg/L PO _4_^3-^ increased to 0.50 ± 0.10 mg/L PO _4_^3-^ indicating nutrient accumulation resulting from agricultural runoff and wastewater discharges that poses eutrophication risks. The ion concentration and buffering capacity of the water bodies showed an upward trend since water conductivity increased from 85 ± 12.3 μS/cm (S1) to 120 ± 12.3 μS/cm (S8) concurrently with alkalinity values rising from 110 ± 10.8 mg/L CaCO _3_ (S1) to 140 ± 10.8 mg/L CaCO _3_ (S8) ( Table 1). Water quality worsens as pollutants from human-related activities continue to escalate downstream.

**Table 1.** Physiochemical parameters of the Ichu rivers across the various sampling sites.

Sampling Site	pH	Turbidity (NTU)	TDS (mg/L)	DO (mg/L)	BOD (mg/L)	Temperature (°C)	Nitrate (mg/L)	Phosphate (mg/L)	Conductivity (μS/cm)	Alkalinity (mg/L)
**S1**	7.9 ± 0.41	2.8 ± 1.95	41.2 ± 5.24	6.3 ± 0.62	14.8 ± 4.61	22.5 ± 0.58	3.2 ± 0.84	0.21 ± 0.10	85 ± 12.3	110 ± 10.8
**S2**	7.6 ± 0.41	3.5 ± 1.95	43.0 ± 5.24	6.0 ± 0.62	17.4 ± 4.61	22.3 ± 0.58	3.5 ± 0.84	0.24 ± 0.10	90 ± 12.3	115 ± 10.8
**S3**	7.4 ± 0.41	4.2 ± 1.95	45.1 ± 5.24	5.7 ± 0.62	19.2 ± 4.61	22.0 ± 0.58	3.8 ± 0.84	0.28 ± 0.10	95 ± 12.3	118 ± 10.8
**S4**	7.2 ± 0.41	5.0 ± 1.95	46.8 ± 5.24	5.5 ± 0.62	21.0 ± 4.61	21.7 ± 0.58	4.2 ± 0.84	0.32 ± 0.10	100 ± 12.3	122 ± 10.8
**S5**	7.1 ± 0.41	5.9 ± 1.95	48.9 ± 5.24	5.2 ± 0.62	23.1 ± 4.61	21.5 ± 0.58	4.5 ± 0.84	0.36 ± 0.10	105 ± 12.3	125 ± 10.8
**S6**	6.9 ± 0.41	6.8 ± 1.95	51.3 ± 5.24	4.9 ± 0.62	25.4 ± 4.61	21.2 ± 0.58	4.9 ± 0.84	0.41 ± 0.10	110 ± 12.3	130 ± 10.8
**S7**	6.8 ± 0.41	7.3 ± 1.95	53.1 ± 5.24	4.7 ± 0.62	27.0 ± 4.61	21.0 ± 0.58	5.2 ± 0.84	0.45 ± 0.10	115 ± 12.3	135 ± 10.8
**S8**	6.7 ± 0.41	7.9 ± 1.95	54.8 ± 5.24	4.5 ± 0.62	28.5 ± 4.61	20.8 ± 0.58	5.5 ± 0.84	0.50 ± 0.10	120 ± 12.3	140 ± 10.8

### Heavy metals concentration in the Ichu River across various sampling sites

Heavy metals displayed an upward trend in concentration from S1 upstream to S8 in the downstream section due to anthropogenic activities, including mining, industrial effluents, and wastewater discharges. The concentrations in the water samples between S1 and S8 spanned from 0.040 ± 0.002 mg/L to 0.072 ± 0.005 mg/L which exceeded WHO's set limit of 0.01 mg/L thus posing substantial health risks. The mining sites and industrial waste facilities appeared to have left their marks with elevated heavy metal content in the S6–S8 regions ( Table 2). The measurement results showed that Lead content in water ranged from 0.014 ± 0.001 mg/L (S1) to 0.032 ± 0.003 mg/L (S8) and exceeded the WHO permitted limit of 0.01 mg/L in all measured areas. Lead contamination results from industrial discharges, battery leakage, and vehicle runoff based on rising analytical data ( Table 2). Cadmium (Cd) concentrations increased from 0.002 ± 0.0003 mg/L (S1) to 0.012 ± 0.0007 mg/L (S8) above the WHO limit (0.003 mg/L) and were most prominent in downstream areas because of electroplating activities, plastic industries, and fertilizer usage. The chromium (Cr) content in the river rose steadily from 0.026 ± 0.002 mg/L at S1 to 0.042 ± 0.004 mg/L at S8, which was near the WHO limit (0.05 mg/L). The elevated level measurements in industrial locations S5 to S8 signify pollution resulting from tanneries and metal industries, alongside waste disposal activities ( Table 2). Heavy metal pollution in the Ichu River has become increasingly serious as we move further downstream because contamination meets or exceeds WHO safety limits, which creates important health and ecological threats.

**Table 2.** Heavy metal concentration in the Ichu River.

Sampling site	Arsenic (mg/L)	Lead (mg/L)	Cadmium (mg/L)	Chromium (mg/L)
S1	0.040 ± 0.002	0.014 ± 0.001	0.002 ± 0.0003	0.026 ± 0.002
S2	0.048 ± 0.003	0.016 ± 0.002	0.003 ± 0.0004	0.028 ± 0.002
S3	0.053 ± 0.003	0.019 ± 0.002	0.005 ± 0.0005	0.031 ± 0.003
S4	0.058 ± 0.004	0.022 ± 0.002	0.006 ± 0.0005	0.034 ± 0.003
S5	0.061 ± 0.004	0.024 ± 0.002	0.008 ± 0.0006	0.036 ± 0.003
S6	0.065 ± 0.004	0.027 ± 0.002	0.009 ± 0.0006	0.038 ± 0.003
S7	0.069 ± 0.005	0.030 ± 0.003	0.011 ± 0.0007	0.040 ± 0.004
S8	0.072 ± 0.005	0.032 ± 0.003	0.012 ± 0.0007	0.042 ± 0.004

### Microbial contamination

**Figure 1.**
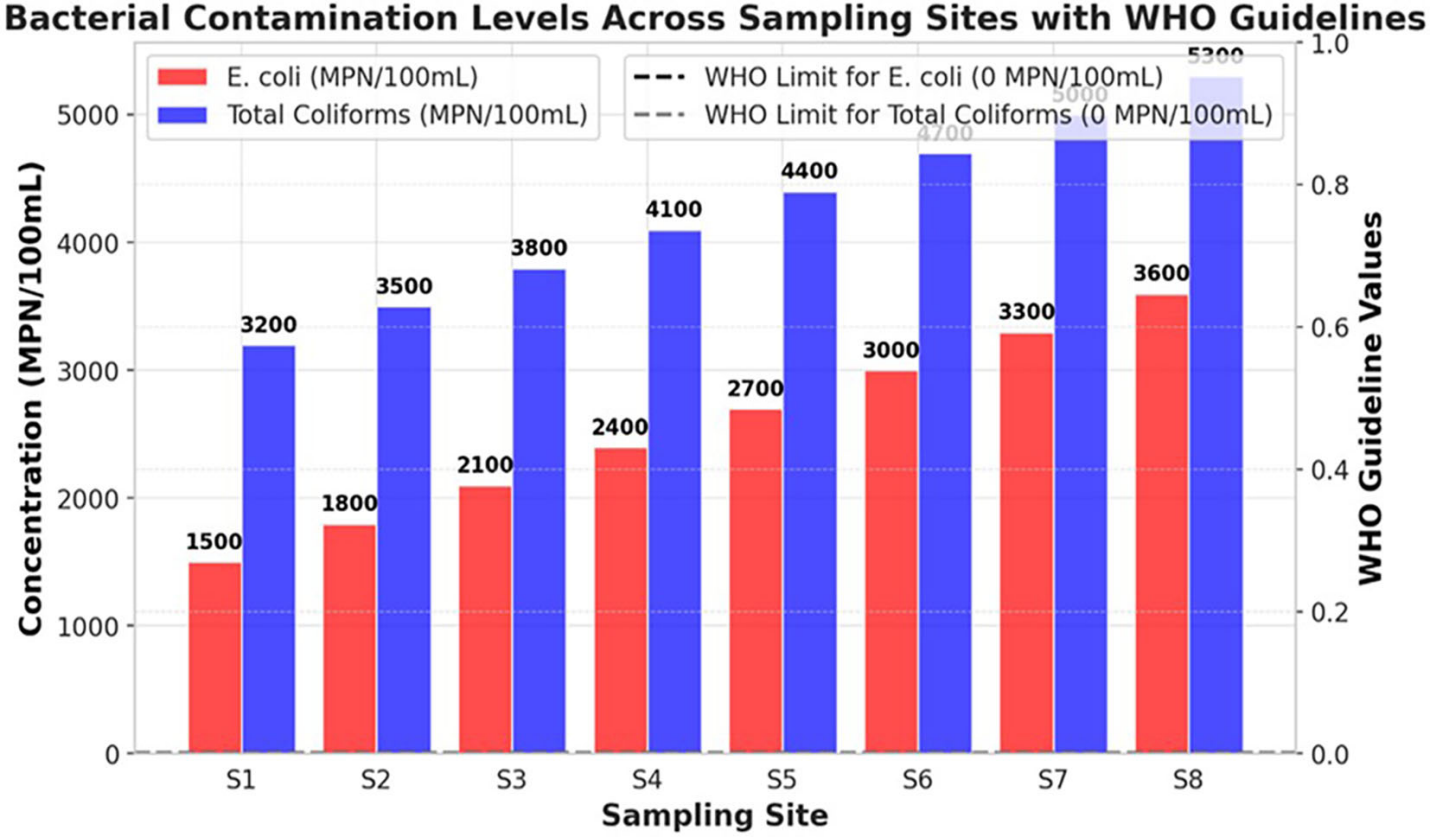
Bacterial contamination levels in the Ichu River.

The bacterial contamination levels of the Ichu River worsened from upstream (S1) to downstream (S8), showing a continuous increase in pollution stemming from human activities. The river demonstrates high pollution levels through fecal and environmental bacterial contamination, because E. coli and Total Coliforms exceed the WHO standard limit of 0 MPN/100mL. This indicates that unhygienic water is unsuitable for direct human consumption ( Figure 1). The bacterial content at S1 showed low levels of contamination because E. coli reached 1500 MPN/100mL while Total coliforms were measured at 3200 MPN/100mL. Bacterial levels experienced substantial growth in the river until they reached the location at S8 where E. coli reached a peak of 3600 MPN/100mL along with Total Coliforms reaching 5300 MPN/100mL. The data showed that microbial contamination originates from sewage outflows, together with runoff from agricultural and industrial facilities that release wastewater. The Total Coliform counts remained higher than the E. coli measurements, indicating that the river water contained bacterial elements from both intestinal sources and general environmental pollution. The rising E. coli numbers at S6–S8 indicate that raw feces entered the water column through unprocessed sewage and livestock waste disposal into the river ( Figure 1). At all measurement points, the Ichu River exceeded the WHO drinking water standards to such an extent that it created a major health threat because individuals exposed to these bacteria risk contracting diarrhea dysentery cholera and typhoid. This study shows an urgent need to implement corrective measures to combat microbial pollution throughout the Ichu River. Three essential measures must be implemented to decrease water pollutant levels and safeguard public wellbeing: a system of improved wastewater treatment, stricter agricultural runoff controls, and effective sanitation management.

### Human health risk assessment

The results indicated a progressive increase in Chronic Daily Intake (CDI), Hazard Quotient (HQ), and Carcinogenic Risk (CR) for both As and Pb from upstream (S1) to downstream (S8), reflecting worsening contamination levels. The CDI for arsenic increased from 0.0012 ± 0.0001 mg/kg/day (S1) to 0.0026 ± 0.0003 mg/kg/day (S8), whereas that for lead increased from 0.0008 ± 0.0001 mg/kg/day (S1) to 0.0015 ± 0.0002 mg/kg/day (S8) ( Table 3). This steady rise suggests increasing anthropogenic pollution, likely from industrial discharge, mining, and wastewater contamination. The HQ values for arsenic range from 4.00 ± 0.30 at S1 to 8.67 ± 0.65 at S8, indicating that exposure at all sites exceeds the non-carcinogenic safety threshold (HQ > 1), posing potential chronic health risks such as neurological and cardiovascular disorders. Similarly, lead HQ values increase from 2.50 ± 0.20 at S1 to 4.60 ± 0.42 at S8, suggesting continuous exposure risks from drinking or using contaminated water. Both metals exhibited HQ values far beyond the acceptable limits, emphasizing the need for immediate intervention. The carcinogenic risk (CR) for arsenic was notably high, exceeding the acceptable risk limit (1E-04) at all sites. The CR values ranged from 6.0E-04 ± 0.5E-05 (S1) to 1.3E-03 ± 1.2E-05 (S8), indicating that long-term exposure to arsenic substantially increased cancer risk. Likewise, CR values, although lower than arsenic, increased from 1.2E-04 ± 0.1E-05 (S1) to 2.6E-04 ± 0.5E-05 (S8), suggesting a growing risk of carcinogenic effects. The higher values in the downstream locations (S6–S8) highlight worsening contamination, requiring urgent mitigation measures. The experimental data in Table 3 show increasing CDI, HQ, and CR values at downstream sampling sites, which can be compared with the recommended values in Table 4 as provided by WHO and USEPA. This comparison highlights deviations from the safety thresholds, emphasizing potential health risks in these areas.

**Table 3.** Health risk assessment across various sampling sites of Ichu River.

Sampling Site	CDI (mg/kg/day) Arsenic	HQ Arsenic	CR Arsenic	CDI (mg/kg/day) Lead	HQ Lead	CR Lead
**S1**	0.0012 ± 0.0001	4.00 ± 0.30	6.0E-04 ± 0.5E-05	0.0008 ± 0.0001	2.50 ± 0.20	1.2E-04 ± 0.1E-05
**S2**	0.0014 ± 0.0001	4.67 ± 0.35	7.1E-04 ± 0.6E-05	0.0009 ± 0.0001	2.80 ± 0.25	1.4E-04 ± 0.2E-05
**S3**	0.0016 ± 0.0001	5.33 ± 0.40	8.2E-04 ± 0.7E-05	0.0010 ± 0.0001	3.10 ± 0.28	1.6E-04 ± 0.2E-05
**S4**	0.0018 ± 0.0002	6.00 ± 0.45	9.3E-04 ± 0.8E-05	0.0011 ± 0.0001	3.40 ± 0.30	1.8E-04 ± 0.3E-05
**S5**	0.0020 ± 0.0002	6.67 ± 0.50	1.0E-03 ± 0.9E-05	0.0012 ± 0.0002	3.70 ± 0.35	2.0E-04 ± 0.3E-05
**S6**	0.0022 ± 0.0002	7.33 ± 0.55	1.1E-03 ± 1.0E-05	0.0013 ± 0.0002	4.00 ± 0.38	2.2E-04 ± 0.4E-05
**S7**	0.0024 ± 0.0002	8.00 ± 0.60	1.2E-03 ± 1.1E-05	0.0014 ± 0.0002	4.30 ± 0.40	2.4E-04 ± 0.4E-05
**S8**	0.0026 ± 0.0003	8.67 ± 0.65	1.3E-03 ± 1.2E-05	0.0015 ± 0.0002	4.60 ± 0.42	2.6E-04 ± 0.5E-05

**Table 4.** World Health Organization (WHO) and the United States Environmental Protection Agency (USEPA) have established maximum allowable limits for arsenic (As) and lead (Pb) in drinking water and human exposure.

Parameter	WHO/USEPA recommended limit
Arsenic (As) CDI	0.0003 mg/kg/day (USEPA)
Arsenic (As) HQ	HQ < 1 (Safe Level)
Arsenic (As) Carcinogenic Risk (CR)	Acceptable limit: 1.0E-04 (USEPA)
Lead (Pb) CDI	0.0004 mg/kg/day (USEPA)
Lead (Pb) HQ	HQ < 1 (Safe Level)
Lead (Pb) Carcinogenic Risk (CR)	Acceptable limit: 1.0E-04 (USEPA)

**Figure 2.**
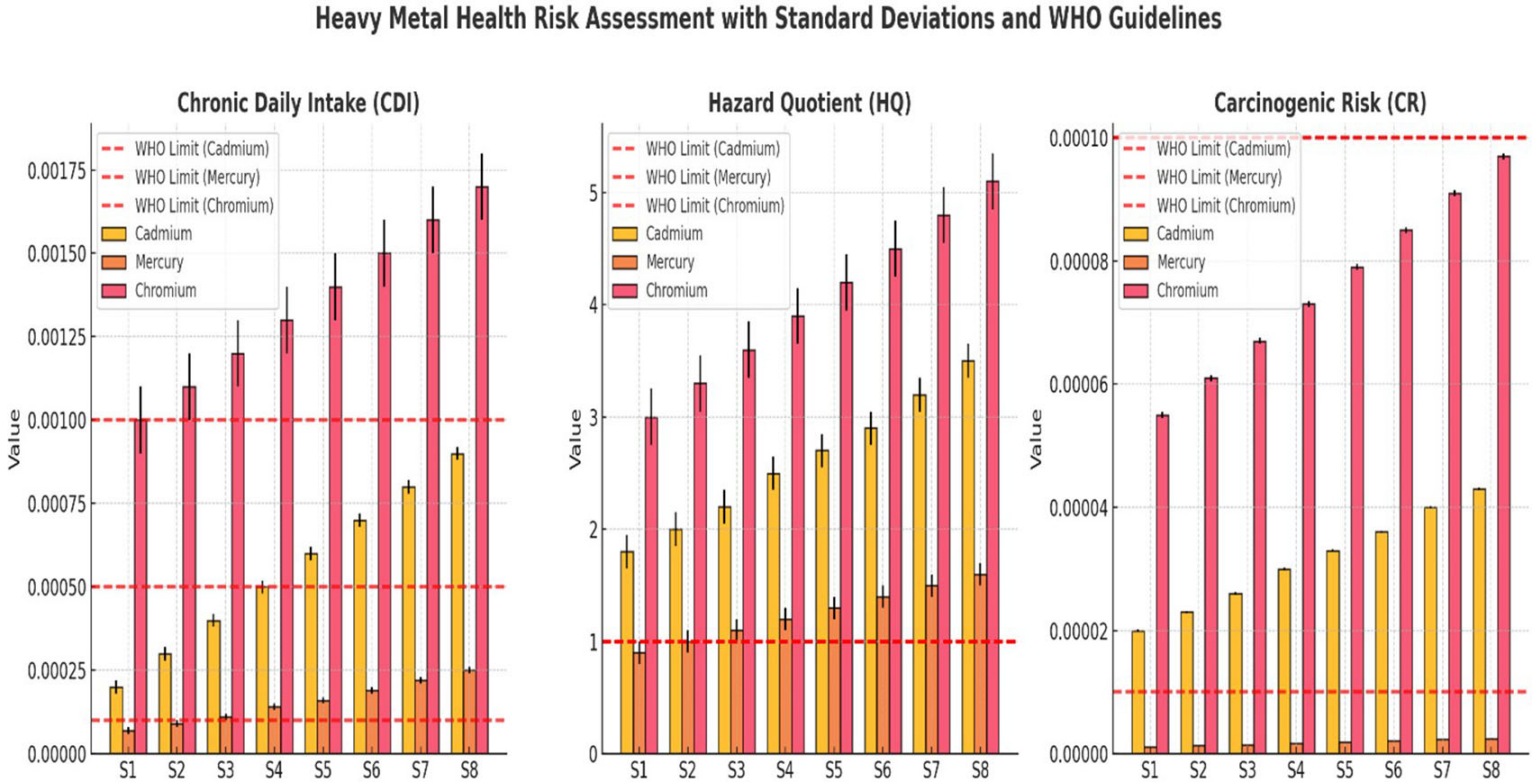
Human health risk assessment along various sites Ichu River water sample.

CDI, HQ, and CR for Cadmium, Mercury, and Chromium across sampling sites (S1–S8) revealed a progressive increase in contamination downstream ( Figure 2). Cr exhibited the highest CDI values, exceeding the WHO limits in S6–S8, while Cd and Hg also showed increasing trends, indicating growing contamination at lower sites. The WHO-recommended CDI thresholds consistently surpassed those of downstream locations. The HQ values demonstrated that Cr and Cd exceeded the non-carcinogenic safety limit (HQ = 1) at all sites, confirming their potential chronic health risks ( Figure 2). Mercury HQ values remained below the critical threshold, but increased steadily downstream, reflecting a rising exposure risk over time. The WHO safety guidelines for HQ were violated for Cr and Cd, highlighting their higher toxicity impact. The CR values indicated that Cr posed the greatest cancer risk, surpassing the acceptable carcinogenic threshold (1E-04) in multiple locations. Cadmium and Mercury also displayed increasing CR values downstream, confirming the elevated health risks for exposed populations. The WHO-recommended carcinogenic limits were exceeded at several sites, particularly in S6–S8, where contamination levels were the most severe. Overall, the results confirmed that heavy metal pollution intensified downstream, with Cr and Cd presenting the most significant health risks. Urgent intervention strategies, including water treatment, pollution control, and regulatory enforcement, are necessary to prevent long-term exposure hazards and safeguard public health.

### Principal component analysis

**Figure 3.**
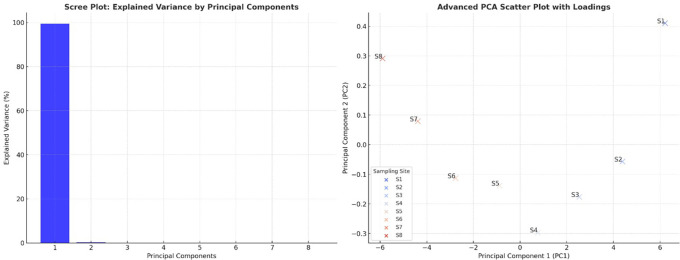
Principal component analysis of water quality parameters.

The scree plot revealed that PC1 explained most of the variance in the dataset, indicating that most water quality parameters were highly correlated and contributed significantly to overall water quality degradation. PC2 accounted for much less variance, suggesting that only a few dominant parameters drove the primary variations in water quality. The sharp drop in variance after PC1 confirmed that the water pollution trends could be effectively summarized using a limited number of components ( Figure 3). The PCA scatter plot demonstrated a clear separation between the upstream and downstream sampling sites (S1–S8), reflecting increasing pollution levels as the river flowed downstream. The upstream sites (S1, S2) appeared distinctly separated from the downstream sites (S7 and S8), reinforcing the notion that water quality deteriorates due to industrial discharge, wastewater inflow, and agricultural runoff. The extreme position of S8 indicated that it had the highest contamination levels, particularly for heavy metals, microbial loads, and nutrient accumulation. Sites S3 to S6 showed a progressive transition, suggesting a gradual decline in water quality, where pollution accumulated and became more severe. The alignment of sites along PC1 suggested that pollution indicators such as heavy metals, coliforms, and organic matter were the primary factors differentiating water quality conditions.

### Correlation matrix

**Figure 4.**
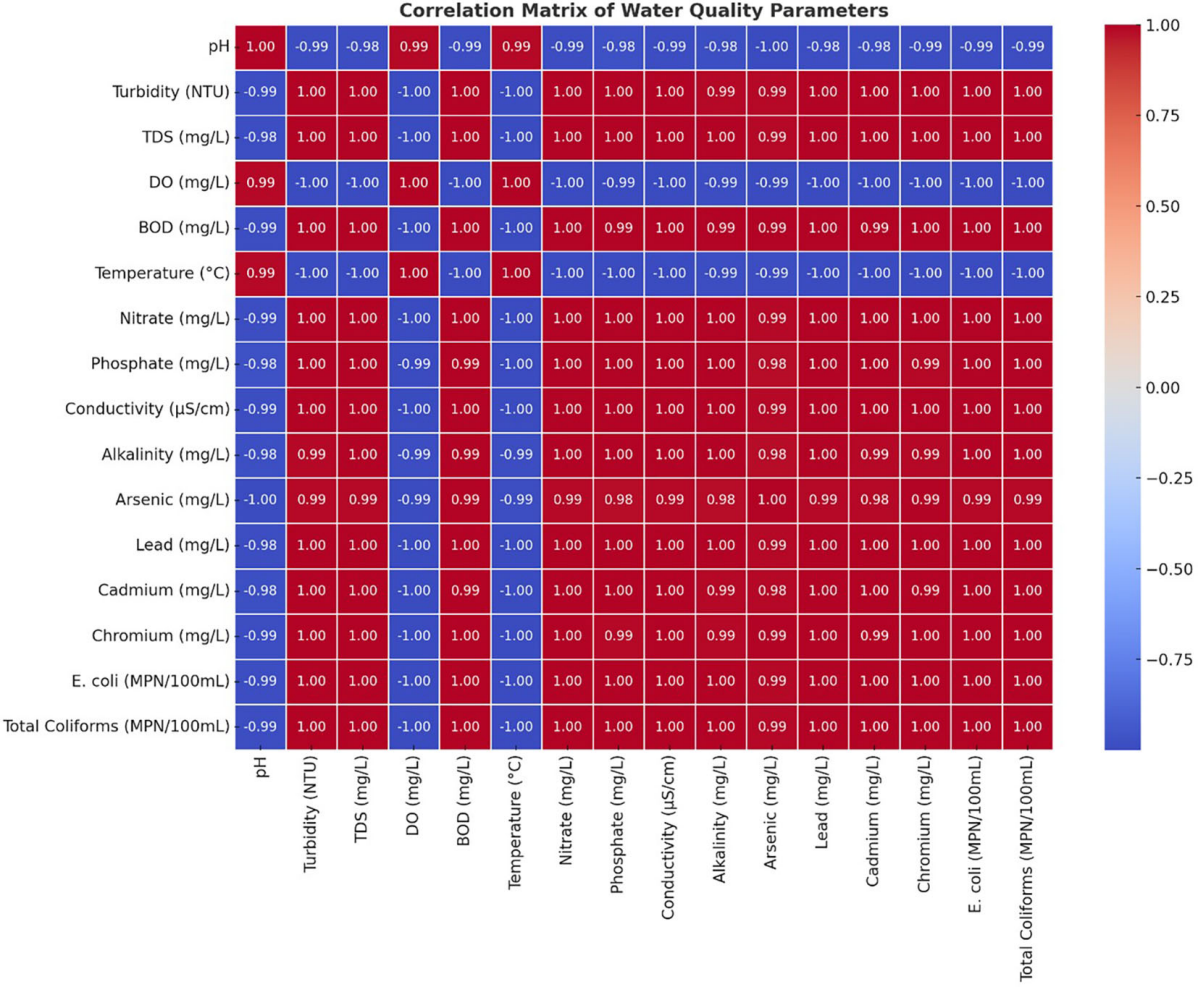
The water quality parameter correlation analysis.

All components of the Turbidity-BOD-Heavy Metal (arsenic-lead-cadmium-chromium)-Microbial Contamination (E. Coli-Total Coliforms) analyses were strongly correlated with each other. Water quality deterioration occurs mainly through industrial effluents, untreated sewage, and agricultural runoff, which carry organic and inorganic contaminants to water sources. The mining sector, along with industries, has substantially elevated both dissolved solids and metal concentrations in rivers by creating high correlations between TDS, Conductivity and Heavy Metals. Scientific analysis showed a clear negative relationship between Dissolved Oxygen readings and all pollution indicators, including BOD, heavy metals, and microbial results ( Figure 4). This study proved that higher pollution concentrations caused the water environment to become dangerous for aquatic organisms. The degradation of water quality worsened because the rising BOD measurements indicated greater organic matter decomposition, which simultaneously reduced the DO levels. The gradual decline of the DO measured downstream matched the rising amounts of waste disposal from industrial and residential sources, which proved the destructive impact of waste emissions. The analysis showed that specific parameters that were strongly associated with each other created redundancy, which made it possible to use important selected variables for monitoring general water quality. Both types of contaminants demonstrated strong associations, which confirmed that they originated from identical sources, such as industrial wastewater streams, urban water runoff, and industrial facilities. The upstream sites S1 and S2 showed decreased indicator correlations, yet the downstream sites S6 through S8 demonstrated increased correlations, indicating that contaminants accumulated during river transport towards the downstream areas.

## Discussion

The water quality analysis starting from S1 to S8 in the Ichu River shows decreasing quality downstream because industrial activities and agricultural and domestic pollution factors intensify. The observed changes in physicochemical measurements confirmed that human activities have a substantial impact on water body health in aquatic environments. Acidification seems to be the cause of the downward shift from 7.9 (S1) to 6.7 (S8) because of industrial waste release and heavy metal pollution. Water quality suffers further deterioration because of turbidity, TDS, and conductivity increase despite sediment transport, surface runoff, and effluent discharge. Previous research has confirmed that rivers located in heavily industrialized and mining areas show identical patterns of environmental decline because of human-caused disturbances ( Giri and Qiu, 2016; Yekta et al., 2023).

The water quality analysis at S1 showed 6.3 mg/L DO but S8 recorded only 4.5 mg/L DO while BOD increased from 14.8 mg/L to 28.5 mg/L. The opposite trend between these variables indicated severe organic contamination at downstream locations, mainly caused by untreated sewage mixed with agricultural runoff and industrial waste products. The sharp rise in BOD levels with decreased DO shows that intense microbial respiration occurs as decomposing organic material and untreated wastewater discharge occurs in numerous heavily polluted river systems ( Yamin et al., 2015). Aquatic life and biodiversity sustain negative effects due to ocean depletion caused by pollution build-up because DO and pollution indicators show a significant negative correlation ( Basant et al., 2010). The degraded water quality has direct socio-economic consequences for local populations, particularly those depending on the river for drinking water and agriculture. Elevated heavy metals and microbial contamination increase the risk of gastrointestinal and chronic illnesses, while also threatening fisheries and food security in riparian communities ( Singh et al., 2024a).

Heavy metals analyzed in the water increased along the downstream direction because of mining operations, improper waste disposal, and industrial discharge. The arsenic levels increased downstream from 0.040 mg/L at S1 to 0.072 mg/L at S8 which surpassed the WHO drinking water standard of 0.01 mg/L creating critical health risks including cancer formation. The analysis revealed that lead mineral substance concentrations doubled from 0.014 mg/L to 0.032 mg/L above the WHO standards, presenting potential neurotoxic hazards. The heavy metal pollutant levels exceeded both national and international standards as Cd increased from 0.010 mg/L to 0.012 mg/L and Cr increased from 0.026 mg/L to 0.042 mg/L. The obtained results match historical findings about rivers adjacent to mining and industrial areas, whose heavy metal contamination stems from tailing ponds alongside electroplating industries and wastewater emissions ( Parida et al., 2021). Our findings are consistent with studies of the Hindon River in India, where high nutrient and heavy metal loads were also reported ( Singh et al., 2024a). Similar contamination has been observed in the Ganges basin ( Li et al., 2022), though regional variations exist due to industrial patterns. This comparative approach strengthens the ecological interpretation of our results. The results of this study support worldwide observations because industrial wastewater and rock dissolution processes act as principal heavy metal pollution pathways in riverine environments ( Zeb et al., 2024; Islam et al., 2018). The microbial tests verified high fecal contamination because total coliform counts increased from 3200 MPN/100mL at S1 to 5300 MPN/100mL at S8, and E. coli numbers rose from 1500 MPN/100mL to 3600 MPN/100mL. The total counts of microorganisms (0 MPN/100mL) set by the WHO exceed what the Ichu River sustains, making it unsafe for human consumption or direct interaction, except through the proper treatment of water. The high detection levels of microbial contaminants in the examined samples were correlated with the effects of urban runoff, untreated sewage, and agricultural waste pollution, in line with previous research on microbial river contamination ( Zahoor and Mushtaq, 2023). A correlation heatmap demonstrated that microbial indicators, together with turbidity and BOD, showed strong positive relationships, which establishes that sewage discharges and organic pollution increase bacterial numbers ( Bowes et al., 2020). The overall water quality trends in this study parallel findings from the Yamuna River in India ( Singh et al., 2021), Bangladesh’s Buriganga River ( Jyethi et al., 2022), and Peru’s Mantaro River ( Choudhury et al., 2022). However, variations in land use, industrial activity, and governance explain differences across these regions, underscoring the need for context-specific solutions.

Assessments of HQ and CR revealed serious safety threats associated with heavy metal exposure. Arsenic HQ levels at S1 measured 4.00 and rose up to 8.67 at S8 thus exceeding the safety threshold (HQ < 1) which suggests a strong probability of developing chronic health problems such as cancer and cardiovascular diseases. An increase in lead levels rose from 2.50 to 4.60 HQ produced substantial neurological disorder risks, mainly affecting children. The CR values exceeded the WHO standards (1E-04) for arsenic and lead, which indicates an unacceptable hazard for developing cancers during long-term exposure. Studies of heavy metal toxicity in polluted rivers have shown similar results with these findings ( Xia et al., 2018). Principal Component Analysis (PCA) showed pollution-related elements to be the fundamental causes of water quality degradation. Heavy metals, microbial contamination, and organic pollution (BOD and turbidity) were the dominant factors in PC1, which explained the most variability. Monitoring data through the PCA scatter plot displayed upstream locations S1 and S2, situated independently from downstream sites S6 through S8, indicating a trend of increasing pollution throughout the watercourse. Downstream water sites cluster due to centralized pollution influences, which also occur in industrialized and urban river systems ( Singh et al., 2005; Yamin et al., 2015. The correlation heatmap confirmed these insights by showing extreme connections between heavy metals and turbidity, and between microbial indicators and BOD, which implies that pollution sources work together as a system. Research worldwide has demonstrated that industrial production, together with urbanization, has adverse effects on freshwater systems ( Ouma et al., 2022).

This study shows that the Ichu River does not meet the water quality criteria defined by the WHO or those set by the USEPA for safe drinking water consumption and protection of aquatic ecosystems. Environmental safety and public health remain at significant risk due to the high levels of heavy metals, microbial contaminants, and organic pollutants that require immediate regulatory action. Authorities should establish a system of strict wastewater treatment regulations while installing advanced heavy metal removal systems through adsorption and coagulation, reverse osmosis, and strengthening the water quality monitoring system. Communities must actively participate in conservation activities, whereas public education programs must teach the public how to conduct proper waste disposal and maintain sanitary conditions. The current study presents significant pollution dynamics knowledge for the Ichu River, but research needs continuous expansion to evaluate water quality changes across seasons and wet and dry periods. Bioaccumulation studies on heavy metals in aquatic organisms must be conducted because they indicate how heavy metals affect fish populations and human food security. Hydrodynamic system simulators and predictive models should be developed to forecast pollution patterns and assist with effective river management practices. Nature-based remediation strategies that use cost-effective techniques for constructed wetlands and phytoremediation offer potential sustainable solutions to combat pollution problems. A key highlight of this study is the combination of physicochemical and biological parameters to provide a comprehensive view of river health. The prospects include developing predictive water quality models and expanding monitoring to evaluate climate change impacts on river ecosystems ( Choudhury et al., 2022; Singh et al., 2024b).

*Prospects and recommendations*

Future work should adopt long-term monitoring programs, advanced statistical and modeling approaches (e.g., multivariate regression, machine learning), and include climate change projections. Policy actions should prioritize improved wastewater treatment, industrial effluent regulation, and community awareness campaigns to reduce water pollution.

*Limitations of the study*

This study is limited by its short-term sampling period, which restricts the ability to capture long-term seasonal fluctuations. Additionally, while multiple water quality parameters were examined, advanced modeling approaches such as machine learning were beyond the current scope. Future studies should address these gaps to provide a more holistic assessment.

## Conclusion

The findings of this study confirm that the Ichu River is experiencing severe water quality degradation owing to increasing industrial, agricultural, and domestic pollution. The progressive rise in turbidity, TDS, conductivity, BOD, and microbial contamination from upstream to downstream sites indicates that anthropogenic activities are the primary drivers of water pollution. The significant decline in DO levels, particularly at downstream sites (S6–S8), highlights the detrimental impact of organic pollution on aquatic ecosystems. The presence of elevated concentrations of heavy metals (As, Pb, Cd, and Cr) in downstream areas exceeding the WHO and USEPA drinking water limits suggests severe contamination from industrial discharge, mining activities, and untreated sewage outflows. The high microbial loads (E. coli and total coliforms), far exceeding the WHO standards, further confirm that the Ichu River is unsuitable for direct human consumption or recreational activities without proper treatment. Given that all key parameters exceed the international water quality standards, urgent intervention is required to prevent long-term environmental and public health crises. Regulatory authorities must implement strict wastewater treatment policies, enforce pollution control measures for industrial discharge, and promote sustainable agricultural practices to reduce contamination levels. Additionally, community engagement, public awareness programs, and regular water quality monitoring should be prioritized to ensure the protection and restoration of the Ichu River. These findings emphasize the need for integrated water resource management strategies to mitigate pollution impacts and safeguard freshwater ecosystems for future generations. This study demonstrates that the river ecosystem is under considerable stress from anthropogenic activities, with direct implications for human health and biodiversity. The outcomes highlight the necessity of integrated watershed management and stricter pollution control policies. The most significant findings include: (i) elevated concentrations of heavy metals exceeding international guidelines, (ii) marked seasonal variation in physicochemical parameters, and (iii) evidence of ecological risk and public health concerns. These findings collectively emphasize the urgent need for mitigation measures.

## Ethics and consent

Ethical approval and consent were not required.

## Data Availability

Zenodo: Raw data of the research paper, Doi: https://doi.org/10.5281/zenodo.14847081 ( Hernández Rodríguez, R. (2025)). This project contains the following underlying data:
• data.xlsx Data is available under the terms of the Creative Commons Attribution 4.0 International

## References

[ref1] Basant N Gupta S Malik A : Linear and nonlinear modeling for simultaneous prediction of dissolved oxygen and biochemical oxygen demand of the surface water—a case study. *Chemom. Intell. Lab. Syst.* 2010;104(2):172–180. 10.1016/j.chemolab.2010.08.005

[ref2] Bowes MJ Read DS Joshi H : Nutrient and microbial water quality of the upper Ganga River, India: identification of pollution sources. *Environ. Monit. Assess.* 2020;192:1–20. 10.1007/s10661-020-08456-2 32691241

[ref3] Brand AS : *Critical evaluation of the accuracy of the enumeration methodology of Coliforms and E. Coli in water from rivers used for the irrigation of fresh produce.* Stellenbosch: Stellenbosch University;2012. Doctoral dissertation.

[ref4] Chigbu P Sobolev D : Bacteriological analysis of water. *Handbook of water analysis.* CRC Press;2007; pp.111–148.

[ref28] Choudhury M Jyethi D Dutta J : Investigation of groundwater and soil quality near to a municipal waste disposal site in Silchar, Assam, India. *Int. J. Energy Water Resour.* 2022;6(1):37–47. 10.1007/s42108-021-00117-5

[ref5] Custodio M Cuadrado W Peñaloza R : Human risk from exposure to heavy metals and arsenic in water from rivers with mining influence in the Central Andes of Peru. *Water.* 2020;12(7):1946.

[ref6] Edo GI Itoje-akpokiniovo LO Obasohan P : Impact of environmental pollution from human activities on water, air quality and climate change. *Ecological Frontiers.* 2024;44:874–889. 10.1016/j.ecofro.2024.02.014

[ref29] Gaur M Singh B Choudhury M : Advances in water quality monitoring and ecological risk assessment under changing climate. *Front. Environ. Sci.* 2022;10:881623. 10.3389/fenvs.2022.881623

[ref7] Giri S Qiu Z : Understanding the relationship of land uses and water quality in Twenty First Century: A review. *J. Environ. Manag.* 2016;173:41–48. 10.1016/j.jenvman.2016.02.029 26967657

[ref8] Hernández Rodríguez R : Raw data from the research paper.[Dataset]. *F1000Research. Zenodo.* 2025. 10.5281/zenodo.14847081

[ref9] Islam MS Proshad R Ahmed S : Ecological risk of heavy metals in sediment of an urban river in Bangladesh. *Hum. Ecol. Risk Assess. Int. J.* 2018;24(3):699–720. 10.1080/10807039.2017.1397499

[ref30] Jyethi DS Yadav AK Siddiqui Z : Source apportionment and risk assessment of trace element pollution in Yamuna river water in Delhi: a probability-based approach. *Urban Water J.* 2022;20(10):1635–1646.

[ref10] Khalef RN Hassan AI Saleh HM : Heavy metal’s environmental impact. *Environmental Impact and Remediation of Heavy Metals.* IntechOpen;2022.

[ref11] Kumi S Adu-Poku D Attiogbe F : Dynamics of land cover changes and condition of soil and surface water quality in a Mining–Altered landscape, Ghana. *Heliyon.* 2023;9(7):e17859. 10.1016/j.heliyon.2023.e17859 37539219 PMC10395293

[ref31] Li L Haoran Y Xiaocang X : Effects of water pollution on human health and disease heterogeneity: A review. *Front. Environ. Sci.* 2022;10:880246. 10.3389/fenvs.2022.880246

[ref12] Muze NE Opara AI Ibe FC : Assessment of the geo-environmental effects of activities of auto-mechanic workships at Alaoji Aba and Elekahia Port Harcourt, Niger Delta, Nigeria. *Environmental analysis, health and toxicology.* 2020;35(2). 10.5620/eaht.e2020005 PMC7374190 32693557

[ref13] Ouma KO Shane A Syampungani S : Aquatic ecological risk of heavy-metal pollution associated with degraded mining landscapes of the Southern Africa River Basins: A review. *Minerals.* 2022;12(2):225.

[ref14] Parida VK Saidulu D Majumder A : Emerging contaminants in wastewater: A critical review on occurrence, existing legislations, risk assessment, and sustainable treatment alternatives. *J. Environ. Chem. Eng.* 2021;9(5):105966.

[ref15] Rahman Z Singh VP : The relative impact of toxic heavy metals (THMs) (arsenic (As), cadmium (Cd), chromium (Cr)(VI), mercury (Hg), and lead (Pb)) on the total environment: an overview. *Environ. Monit. Assess.* 2019;191:1–21. 10.1007/s10661-019-7528-7 31177337

[ref16] Sánchez-Araujo V Portuguez-Maurtua M Palomino-Pastrana P : Water quality index and health risks in a peruvian high andean river. *Ecological Engineering & Environmental Technology.* 2024;25:301–315. 10.12912/27197050/187227

[ref17] Saxena V : Water Quality, Air Pollution, and Climate Change: Investigating the Environmental Impacts of Industrialization and Urbanization. *Water Air Soil Pollut.* 2025;236(2):1–40. 10.1007/s11270-024-07702-4

[ref18] Singh KP Malik A Sinha S : Water quality assessment and apportionment of pollution sources of Gomti river (India) using multivariate statistical techniques—a case study. *Anal. Chim. Acta.* 2005;538(1-2):355–374. 10.1016/j.aca.2005.02.006

[ref25] Singh B Choudhury M Samanta P : Ecological risk assessment of heavy metals in adjoining sediment of river ecosystem. *Sustainability.* 2021;13(18):10330. 10.3390/su131810330

[ref26] Singh B Choudhury M Gupta P : Physicochemical and biological characteristics of River Hindon at Galheta station from 2009 to 2020. *Environ. Qual. Manage.* 2024a;33(3):331–344. 10.1002/tqem.22115

[ref27] Singh B Samanta P Choudhury M : Physicochemical and heavy metal pollution level in Hindon River ecosystem: An implication to public health risk assessment. *Environ. Qual. Manage.* 2024b;34(2):e22263. 10.1002/tqem.22263

[ref33] Su C Wang Y Zhang H : Assessment of heavy metal contamination and human health risk in drinking water sources: A case study from a rural region in China. *Environ. Sci. Pollut. Res.* 2021;28(34):46852–46865.

[ref32] TQEM : Potential health hazard of drinking water in restaurants and tea stalls. 2023. Reference Source

[ref19] Xia F Qu L Wang T : Distribution and source analysis of heavy metal pollutants in sediments of a rapid developing urban river system. *Chemosphere.* 2018;207:218–228. 10.1016/j.chemosphere.2018.05.090 29800822

[ref20] Yamin M Nasir A Sultan M : Impact of sewage and industrial effluents on water quality in Faisalabad, Pakistan. *Advances in Environmental Biology.* 2015;9(18):53–59.

[ref21] Yekta NM Rafati M Karimi A : Restoration of urban rivers with water quality modeling approach (Case Study: Kan River, Tehran City, Iran). 2023.

[ref22] Yin Z Song J Liu D : Mapping mining-affected water pollution in China: Status, patterns, risks, and implications. *Hydrol. Earth Syst. Sci. Discuss.* 2024;2024:1–35.

[ref23] Zahoor I Mushtaq A : Water pollution from agricultural activities: A critical global review. *Int. J. Chem. Biochem. Sci.* 2023;23(1):164–176.

[ref24] Zeb M Khan K Younas M : A review of heavy metals pollution in riverine sediment from various Asian and European countries: Distribution, sources, and environmental risk. *Mar. Pollut. Bull.* 2024;206:116775. 10.1016/j.marpolbul.2024.116775 39121593

