# Supplementary material for: Integrated water quality assessment and health risk analysis of heavy metal and microbial contamination in the Ichu River, Peru
Source: F1000Res. 2026 Jan 31;14:384. Originally published 2025 Apr 2. [Version 4] doi: 10.12688/f1000research.162022.4 (PMC12869023; doi:10.12688/f1000research.162022.4)
Supplement: Peer review history

# Reviewer report: Reviewer response for version 4

**DOI:** 10.5256/f1000research.195355.r454223

**Published:** 2026-02-03

**Authors:** Moharana Choudhury

**Affiliations:** 1 Environmental Research and Management Division, Voice of Environment (VoE), Guwahati, Assam, India

Now this paper has been improved so it can proceed for acceptance.

 Best wishes

Is the work clearly and accurately presented and does it cite the current literature?

Partly

If applicable, is the statistical analysis and its interpretation appropriate?

Partly

Are all the source data underlying the results available to ensure full reproducibility?

Yes

Is the study design appropriate and is the work technically sound?

Yes

Are the conclusions drawn adequately supported by the results?

Yes

Are sufficient details of methods and analysis provided to allow replication by others?

Yes

Reviewer Expertise:

Now this paper has been improved.

I confirm that I have read this submission and believe that I have an appropriate level of expertise to confirm that it is of an acceptable scientific standard.

# Reviewer report: Reviewer response for version 2

**DOI:** 10.5256/f1000research.188295.r424942

**Published:** 2025-11-25

**Authors:** Zeid A. ALOthman

**Affiliations:** 1 King Saud University, Riyadh, Riyadh Province, Saudi Arabia

**Comments and suggestions:**

**1. Abstract: **“The Ichu River serves as the primary water source for urban and agricultural use and industrial operations, but anthropogenic pollution has a serious negative impact on its water quality.” – Add more text to explain the background.

 2. “Keywords: Use more suitable different keywords which are not mentioned in title of the manuscript.

 3. “Water quality assessment, combined with health risk evaluation for river contaminants, must be performed because of the region's economic reliance on this water source.” --- The authors need to discuss a paragraph related application of various materials in environmental applications for metal analysis. They can check the below related references, which may improve the supporting information.

 Colloids and Surfaces A: Physicochemical and Engineering Aspects 647, 2022, 129077

 Journal of Colloid and Interface Science 646, 2023, 129-140

 Electrochimica acta 386, 2015, 138482

 Chemical Engineering Journal 270, 2015, 9-21

 Separation Science and Technology 55 (10), 2020, 1766-1775

 Desalination and Water Treatment 57 (46), 2016, 21863-21869

 4. “All sampling site locations have been provided below as S1, S2, S3,…,.” -What basic condition were selected for sample collection?

 5. “The analysis focused on measuring arsenic (As), lead (Pb), cadmium (Cd), mercury (Hg), and chromium (Cr) in water samples using Inductively Coupled Plasma Mass Spectrometry (ICP-MS, Agilent 7500 Series, USA).” – Provide details for each instrument used including city and country.

 6. “Heavy metals analyzed in the water increased along the downstream direction because of mining operations, improper waste disposal, and industrial discharge.” - How this was concluded, explain with supportive information.

 7. “Table 1. Physiochemical parameters of the Ichu rivers across the various sampling sites.” – Prepare all tables in three-lines format.

 8. “Figure 1. Bacterial contamination levels in the Ichu River.”  – Add optimal conditions in each figure.

 9. All the typos and grammar need to check thoroughly in the manuscript.

Is the work clearly and accurately presented and does it cite the current literature?

Yes

If applicable, is the statistical analysis and its interpretation appropriate?

I cannot comment. A qualified statistician is required.

Are all the source data underlying the results available to ensure full reproducibility?

Partly

Is the study design appropriate and is the work technically sound?

Yes

Are the conclusions drawn adequately supported by the results?

Partly

Are sufficient details of methods and analysis provided to allow replication by others?

Yes

Reviewer Expertise:

Analytical Chemistry

I confirm that I have read this submission and believe that I have an appropriate level of expertise to confirm that it is of an acceptable scientific standard.

# Reviewer report: Reviewer response for version 1

**DOI:** 10.5256/f1000research.178144.r375345

**Published:** 2025-05-09

**Authors:** Moharana Choudhury

**Affiliations:** 1 Environmental Research and Management Division, Voice of Environment (VoE), Guwahati, Assam, India

This paper is good, but it needs some improvements.

 To give the present study a proper scientific base and justification, add more citations/references based on recent scientific research on the topic worldwide and in the region. 

 In the introduction section, add more information on recent research studies on water quality and its implications on global and regional levels.

 Add some case studies worldwide and in the region to highlight the significance of this current study.

 What are the principal significances of this study? Please highlight

 What are the significant limitations of this study?

 What are the new highlights and prospects of this current study?

 Please add/ highlight the impacts on local people.

 Please add more models, water index, or information to make it more scientifically significant.

 Strengthen the discussion section.

 Add figures/photos to the illustrations in the study area and make them relevant.

 What are the significant findings or highlights of this study? Please add on MS. 

 Improve the conclusion part with the significant outcome of the study

 Add a section before the conclusion containing future perspectives and recommendations from this current study.

 Justify the overall water quality and river ecosystem with global and regional comparisons (other countries/Regions).  

 The authors can refer to these papers to add statistical and modeling-related input, which will help to improve the overall paper's technical and scientific quality.

**https://doi.org/10.1007/s42108-021-00117-5**

**https://doi.org/10.3390/su131810330**

**https://doi.org/10.3389/fenvs.2022.881623**

**https://doi.org/10.1002/tqem.22115**

**https://doi.org/10.1002/tqem.22263**

 These will make the paper a good one and good to go for indexing.

 Best wishes

Is the work clearly and accurately presented and does it cite the current literature?

Partly

If applicable, is the statistical analysis and its interpretation appropriate?

Partly

Are all the source data underlying the results available to ensure full reproducibility?

Yes

Is the study design appropriate and is the work technically sound?

Yes

Are the conclusions drawn adequately supported by the results?

Yes

Are sufficient details of methods and analysis provided to allow replication by others?

Yes

Reviewer Expertise:

Need overall improvements for this MS

I confirm that I have read this submission and believe that I have an appropriate level of expertise to confirm that it is of an acceptable scientific standard, however I have significant reservations, as outlined above.

## References

[1] : Ecological Risk Assessment of Heavy Metals in Adjoining Sediment of River Ecosystem. *Sustainability* .2021;13(18) : 10.3390/su131810330 10.3390/su131810330

[2] : Investigation of groundwater and soil quality near to a municipal waste disposal site in Silchar, Assam, India. *International Journal of Energy and Water Resources* .2022;6(1) : 10.1007/s42108-021-00117-5 37-47 10.1007/s42108-021-00117-5

[3] : Physicochemical and biological characteristics of River Hindon at Galheta station from 2009 to 2020. *Environmental Quality Management* .2024;33(3) : 10.1002/tqem.22115 331-344 10.1002/tqem.22115

[4] : Physicochemical and heavy metal pollution level in Hindon River ecosystem: An implication to public health risk assessment. *Environmental Quality Management* .2024;34(2) : 10.1002/tqem.22263 10.1002/tqem.22263

# Response

**Published:** 2026-01-15

**Authors:** RUSSBELT YAULILAHUA HUACHO

**Affiliations:** Huancavelica, Universidad Nacional de Huancavelica, Huancavelica, Huancavelica, Peru

**General comment**

 This paper is good, but it needs some improvements.

**Answer:** We thank the reviewer for this valuable suggestion. Accordingly, we strengthened the scientific justification of the study by adding recent (2021–2025) global, regional, and Peruvian/Andean literature to the Introduction and Discussion, including worldwide and regional case studies on river water quality, heavy metal and microbial contamination, and health risk assessment. Additional references were also incorporated to support the use of water quality indices and integrated assessment approaches, thereby enhancing the manuscript’s scientific rigor and relevance.

**Comment 1:** To give the present study a proper scientific base and justification, add more citations/references based on recent scientific research on the topic worldwide and in the region. 

**Answer 1:** We appreciate the reviewer’s suggestions and have addressed them by clearly highlighting in red font.

**Comment 2:** In the introduction section, add more information on recent research studies on water quality and its implications on global and regional levels.

**Answer 2:** we expanded the Introduction by adding recent global and regional literature (2021–2025) on river water quality degradation and its ecological and public health implications, with particular emphasis on Andean and Peruvian river systems.

**Comment 3:** Add some case studies worldwide and in the region to highlight the significance of this current study.

**Answer 3: **Suggested changes have been made as suggested by the reviewer. Kindl see the revised of the paper.

**Comment 4:** What are the principal significances of this study? Please highlight

**Answer 4: **Suggested changes have been made as suggested by the reviewer. Kindl see the revised of the paper.

**Comment 5:** What are the significant limitations of this study?

**Answer 5: **Suggested changes have been made as suggested by the reviewer. Kindl see the revised of the paper.

**Comment 6:** What are the new highlights and prospects of this current study? Please add/ highlight the impacts on local people.

**Answer 6: **Suggested changes have been made as suggested by the reviewer. Kindl see the revised of the paper.

**Comment 7:** Please add more models, water index, or information to make it more scientifically significant.

 Strengthen the discussion section.

**Answer 7: **We enhanced the scientific significance of the study by incorporating additional water quality indices and modeling perspectives in the Methods and by strengthening the Discussion with global–regional comparisons, mechanism-based interpretation, and management-oriented modeling relevance.

**Comment 8:** Add figures/photos to the illustrations in the study area and make them relevant.

**Answer 8: **Suggested changes have been made as suggested by the reviewer. Kindl see the revised of the paper.

**Comment 9:** What are the significant findings or highlights of this study? Please add on MS. 

**Answer 9:** We added a dedicated subsection highlighting the key findings of the study, summarizing major physicochemical trends, heavy metal and microbial contamination, health risk outcomes, and multivariate insights.

**Comment 10:** Improve the conclusion part with the significant outcome of the study

**Answer 10:** The Conclusion was revised to emphasize the most significant outcomes of the study, including upstream–downstream degradation patterns, heavy metal and microbial exceedances, quantified health risks, and their implications for ecosystem integrity and public health.

**Comment 11:** Add a section before the conclusion containing future perspectives and recommendations from this current study.

**Answer 11:** We added a new section titled *“Future perspectives and recommendations”* before the Conclusion, outlining research priorities, advanced modeling prospects, and practical management actions derived from the study’s findings.

**Comment 12:** Justify the overall water quality and river ecosystem with global and regional comparisons (other countries/Regions). 

**Answer 12:** We strengthened the Discussion by adding a dedicated comparison of the Ichu River’s water quality and ecosystem condition with well-documented global and regional river systems, demonstrating that the observed degradation aligns with internationally reported patterns in urban- and mining-impacted rivers.

**Comment 13:** The authors can refer to these papers to add statistical and modeling-related input, which will help to improve the overall paper's technical and scientific quality.

**https://doi.org/10.1007/s42108-021-00117-5**

**https://doi.org/10.3390/su131810330**

**https://doi.org/10.3389/fenvs.2022.881623**

**https://doi.org/10.1002/tqem.22115**

**https://doi.org/10.1002/tqem.22263**

**Answer 13: **Suggested changes have been made as suggested by the reviewer. Kindl see the revised of the paper.

**Comment 14:** These will make the paper a good one and good to go for indexing.

**Answer 14: **Thank you very much for your valuable comments for improving the quality of the paper.
